# Catalytic Oxidation Process for the Degradation of Synthetic Dyes: An Overview

**DOI:** 10.3390/ijerph16112066

**Published:** 2019-06-11

**Authors:** Rahat Javaid, Umair Yaqub Qazi

**Affiliations:** 1 Renewable Energy Research Center, Fukushima Renewable Energy Institute, National Institute of Advanced Industrial Science and Technology, AIST, 2-2-9 Machiikedai, Koriyama, Fukushima 963-0298, Japan; 2 Chemistry Department, College of Science, University of Hafr Al Batin, P.O Box 1803 Hafr Al Batin 31991, Saudi Arabia; umairqazi@uhb.edu.sa

**Keywords:** advanced oxidation process, catalyst, fenton reaction, hydroxyl radical, sulphate radical, synthetic dyes, tubular reactors

## Abstract

Dyes are used in various industries as coloring agents. The discharge of dyes, specifically synthetic dyes, in wastewater represents a serious environmental problem and causes public health concerns. The implementation of regulations for wastewater discharge has forced research towards either the development of new processes or the improvement of available techniques to attain efficient degradation of dyes. Catalytic oxidation is one of the advanced oxidation processes (AOPs), based on the active radicals produced during the reaction in the presence of a catalyst. This paper reviews the problems of dyes and hydroxyl radical-based oxidation processes, including Fenton’s process, non-iron metal catalysts, and the application of thin metal catalyst-coated tubular reactors in detail. In addition, the sulfate radical-based catalytic oxidation technique has also been described. This study also includes the effects of various operating parameters such as pH, temperature, the concentration of the oxidant, the initial concentration of dyes, and reaction time on the catalytic decomposition of dyes. Moreover, this paper analyzes the recent studies on catalytic oxidation processes. From the present study, it can be concluded that catalytic oxidation processes are very active and environmentally friendly methods for dye removal.

## 1. Introduction

With an increasing world population, the demand for basic raw materials and concluding products is increasing exponentially. Global economic growth and industrial revolutions lead to speedy metropolitanization. According to an estimation, over 700 emerging pollutants such as the waste contaminants of petrochemicals, personal care, textile, and pesticides are being confirmed in the aquatic ecosystem of the European region [1,2,3,4]. Among these, the textile and dyeing industries are considered among the major sources of water contamination. In textile mills, two principal processes contribute to the release of dyes in the environment: one is from the cleaning of the dye tank following the preparation of the dye bath, and the other is from the draining of the dye bath after the dyeing process is complete [5]. Dyeing industries use large volumes of water and dyes. Approximately 8–20% unutilized dyes and auxiliary chemicals are discharged into the wastewater stream from textile industries [6]. Approximately 1–10% of pigments used in paper and leather industries are lost as waste. Thus, tons of dyes are discharged daily into the environment as aquatic waste [7]. The wastewater from these industries contains high levels of biochemical oxygen demand (BOD) and chemical oxygen demand (COD) [8,9]. The discharge of contaminated effluent without any treatment into the environment creates various environmental threats, including depressed photosynthesis and aquatic plant demise [10]. Moreover, most of these dyes and their components are carcinogenic and mutagenic with harmful impacts on all living beings on the earth [11,12,13]. Even the presence of a small quantity of these compounds (less than 1 ppm) in water has adverse effects. Many countries have strict regulations on the release of wastewaters from textile industries without proper processing to remove the excessive concentrations of color and COD [14].

Synthetic dyes are more resistant and difficult to degrade completely using photolysis, biological and chemical decomposition, and other ordinary approaches. Different technologies are accessible to decrease the absorption of these dyes into the environment such as ion exchange, chemical sedimentation, electrochemical reduction, membrane process, and absorption [15,16,17]. All these available technologies have limitations, including the inefficient mineralization of the synthetic dyes, dense solution disposal, high energy consumption, high operation costs, and the excessive production of sludge, etc. [18,19,20]. Therefore, there are intensive demands for highly efficient and progressive newer technologies for the complete removal of the contaminants from the aquatic environment [21,22]. Among new alternative technologies for wastewater treatment, one effective technique is advanced oxidation processes (AOPs), which can be used to convert toxic and resistant chemicals into environmentally benign minerals. The highly efficient degradation of wastewater compounds is achievable by adopting a direct oxidation approach, but the requirement of severe operation conditions (e.g., high temperature and pressure) for the degradation of selected compounds increases the overall process cost [23,24,25,26,27]. Catalytic oxidation is one of the most efficient AOPs, based on the active radicals produced during the reaction in the presence of a catalyst at relatively mild reaction conditions. The catalytic process is based on the formation of strong oxidizing radicals that have dominant abilities to eliminate most of the pollutants present in wastewater. The main objective of the catalytic oxidation process is the conversion of synthetic dyes to benign products [28]. In catalysis, since the processes are performed on the surface of the catalyst, density, porosity, surface area, higher activity and selectivity for an oxidizing radical generation, stability, homogeneity, and low cost become the pivotal factors that affect the overall reaction performance.

This review paper addresses the problems of synthetic dyes and various catalytic degradation techniques available for their efficient mineralization. The main focus of this study is to discuss current research progress and general surveys either in the form of new process developments or the improvement of already existing technologies to accomplish the removal of synthetic dyes by adopting a catalytic oxidation route. Recent developments in the hydroxyl radical-based oxidation process are evaluated, and a critical analysis of various Fenton processes, possible mechanistic approaches, feasible conditions, the effect of various factors, advantages, and disadvantages are summarized. Non-iron metal catalysts and the application of thin metal catalyst-coated tubular reactors have been described in detail. In addition, the sulfate radical-based catalytic oxidation technique has also been summarized.

## 2. Toxic Effects of Dispersed Synthetic Dyes

A dye is a colored organic substance with a common property to absorb visible light and ability to attach strongly with fiber by means of chemical or physical bonding between the groups of fiber and dyes. Commercially, dyes should be quickly responsive to visible light, rubbing, and water. Not all color substances are dyes or dyestuff because color is a physiological perception concerned with light wavelengths hitting the retina of our eyes. The perception of color is only formed when a molecule absorbs the specific wavelength of light in the visible region of the electromagnetic spectrum and transmits or reflects the other wavelengths [29]. Dye molecules are made up of two main components—chromophores and auxochromes. The presence of chromophores in the structure is responsible for color formation while auxochromes work as an additive and make the molecule soluble in water as well as develop a strong attachment with fibers [30,31,32]. Hence, thousands of different dyes are synthesized for potential commercial applications by the alteration in molecular structure. Generally, dyes can be classified based on their chemical structure as well as the existence of specific chromophores. 

**Figure 1:**
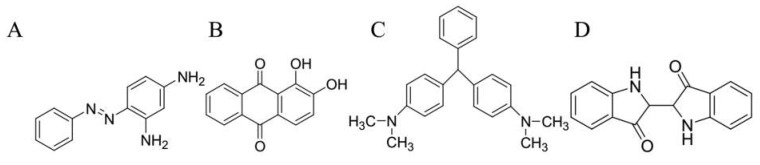
The fundamental structure of some synthetic dyes. (**A**) Azo dye (chrysoidine), (**B**) anthraquinone (alizarine), (**C**) triphenylmethane (malachite green), (**D**) indigo dye (indigo) [33].

The dyes are made up of the by-products of petroleum and the minerals. Various kinds of synthetic dyes are commonly used industrially and classified as azo dyes, anthraquinone, triphenylmethane, phthalocyanine, indigo, and sulfur dyes. Most often, the fundamental chemical structure contains chromophores such as –C=C–, –C=O, –N=N­–, –NO_2_, –C=N, and quinonoid structural rings which are authoritative for the absorption of light in the wavelength of visible range. The classification of each compound is related to the existence of a particular functional group attached with the fundamental structure known as auxochromes, such as halogens, –CO_2_H, –COR, –SO_3_H, –CH_3_CO–, –CH_3_, and –NH_2_ [33]. Figure 1 presents the fundamental structures of some synthetic dyes.

Over 10,000 different types of commercial pigments and dyes are produced annually [34]. Because of their variety of colors, easy utilization, and good stability, synthetic dyes are applied more frequently in industries [35]. The adoption of synthetic dyes spread during the industrial revolution and became a crucial part of the textile, paper, and food industries [36]. The annual production of dyes is reported as over 900,000 metric ton [37], and most of them are used in the textile industry. Over 70% of dyes are synthetic and sold out with their common or commercial names [38,39]. This could be the reason that most of the workers are not aware of their fundamental chemistry and ready to face the severe toxic effects due to the lack of proper handling for degradation. Every year, textile industries discharge an enormous quantity of colored substances into neighboring water without proper treatment, causing major environmental pollution. The industrial revolution means the construction of more industry with the usage of a huge quantity of dyes, increasing the toxicity in the whole ecosystem. Synthetic dyes keep their xenobiotic and wayward nature, resulting in an extensive toxic effect on life. Textile industrial discharge contains a large amount of synthetic dyes along with toxic metal contents which enhance the BOD, COD, and the pH of the surrounding water resources [40,41]. When the dye-containing wastewater is mixed with clean water, it unbalances the recommended level of organic and inorganic parameters. Mixing colored material into the water decreases sunlight penetration deep into the water and affects the whole water ecosystem. The existing toxic compounds of synthetic dyes in water are absorbed by fish and all other living animals in the water. When human eat these poisonous fish, they become affected by the toxic substances, causing many diseases such as cramps, mental disorder, hypertension, etc. Synthetic dyes containing benzidine-based structures are reported as carcinogenic, causing a severe toxic effect on human bladder [42]. Synthetic dyes can easily dissolve in water and penetrate the skin, causing allergic reactions, cancer, and eye irritation [42]. Table 1 summarizes various synthetic dyes and their hazardous effects.

## 3. Fenton Reaction

H.J. Fenton was the first to report the famous Fenton reaction in 1894 and illustrated the oxidation process utilizing hydrogen peroxide (H_2_O_2_) as oxidant and iron (Fe) as a catalyst in the presence of acidic (H^+^) medium [71]. The chemistry of the Fenton reaction has been explained comprehensively in many published review articles [72,73]. The reaction mechanism of the Fenton oxidation reaction is a little complex [74], and various parameters influence the efficiency of the overall process. In general, the Fenton oxidation process starts with the generation of a hydroxyl free radical (**^∙^**OH) [75,76]. Hydroxyl radicals are one of the most active oxidants and can react 10^6^–10^12^ times faster than ozone depending on the substrate to be degraded [77,78,79]. The steps involved in the Fenton reaction are described as below (Equations (1)–(7)), and Equation (1) is the chain initiation process [80,81].

Fe^2+^ + H_2_O_2_ → Fe^3+^ + **^∙^**OH + OH^−^ → [Chain initiation process] (1)

Fe^3+^ + H_2_O_2_ → Fe-OOH^2+^ + H^+^ (2)

Fe-OOH^2+^ → Fe^3+^ + **^∙^**O_2_H (3)

Fe^3+^ + **^∙^**O_2_H → Fe^2+^ + O_2_ + H^+^ (4)

Fe^2+^ + **^∙^**OH → Fe^3+^ + OH^−^ [Chain termination process] (5)

H_2_O_2_ + **^∙^**OH → H_2_O + **^∙^**O_2_H (6)

Organic toxic waste + **^∙^**OH → Degraded products (7)

An acidic medium is required for the generation of hydroxyl radicals, and pH 3 is usually considered as the optimum condition for the Fenton oxidation reaction [82,83,84,85,86]. The formation of a large amount of ferric hydroxide precipitation is observed above pH 4, which decreases the efficiency of the process for dye degradation [87].

The Fenton reaction can be classified into two broad categories—homogeneous and heterogeneous processes. In homogeneous processes, iron species are in the same phase as the reactants and there is no limitation for mass transfer. Sludge formation with high iron contents, the deactivation of iron because of complex formation and a specific pH range (2.0–4.0) dependency are considered as the significant shortcomings of the homogeneous process. All these drawbacks can be conquered by the functionalization of the heterogeneous catalytic approach [88,89]. In heterogeneous catalysis, iron is sustained within the catalytic structure and can efficiently stimulate the degradation of recalcitrant materials without the formation of ferric hydroxide sludge. Based on the current research progress and investigations, three feasible mechanistic routes have been suggested to illustrate the heterogeneous catalytic Fenton reactions [90,91,92,93,94,95,96]. According to the first route, iron is percolated to the reaction solution and stimulates H_2_O_2_ using a homogeneous pathway. Another approach is chemisorption of the investigative molecules on the surface of the catalyst, whereas the third approach is the decomposition of H_2_O_2_ into hydroxyl radicals. Recent research progress reports have shown that heterogeneous catalysis is more competent than homogeneous catalysis for the degradation of synthetic dyes in wastewater [97]. Additionally, heterogeneous catalysis is more beneficial as: (1) the catalyst is easy to use and store, precisely recovered, and has the feasibility to reuse, (2) can be used at a wide range of pHs, and (3) can avoid the generation of ferric hydroxide precipitation. To enhance reaction performance, complexing agents such as nafion, zeolite, activated charcoal, clay, resin, silica have been used as supporting material for Fe [98,99,100,101,102,103,104,105,106]. Various types of Fenton processes are accessible to use such as photo-Fenton, electro-Fenton, photo-electro Fenton, sono-Fenton, sono-photo Fenton, and sono-electro Fenton processes [107,108]. Some of these are briefly described below.

### 3.1. Photo-Fenton Process

The improved form of the conventional Fenton oxidation reaction in the presence of UV–visible light below 600 nm wavelength is called the photo-Fenton reaction [109]. The involvement of UV–visible light provides two additional routes for the release of hydroxyl radicals, enhancing the degradation rate of dye pollutants [110]: (i) the photoreduction of Fe^3+^ to Fe^2+^ ions as shown in Equation (8) [111,112] and (ii) peroxide photolysis via shorter wavelengths (Equation (9)). The photo-generated ferrous ions enter the Fenton reaction to produce hydroxyl radicals. Therefore, the oxidation rate of the photo-Fenton process is higher than the Fenton process without using UV–visible light [113]. Moreover, the iron utilization and resultant sludge formation are comparably much reduced in photo-Fenton reaction [114].

[Fe(OH)]^2+^ + *h*υ → Fe^2+^ + **^∙^**OH; λ < 580 nm (8)

H_2_O_2_ + *h*υ → 2 **^∙^**OH; λ < 310 nm (9)

In recent years, several reviews have been published on the applications of photo-Fenton processes for the removal of various kinds of organic pollutants present in wastewater [115,116]. The researchers have described many factors affecting the performance of the photo-Fenton process, including the type of light source, the power of the lamp, the structure of the reactor, metal concentration and H_2_O_2,_ etc. Among these factors, power and the source of light play an important role in determining the efficiency of the reaction [117,118]. Conventional UV lamps are used as a light irradiation source available as low-, medium-, and high-pressure mercury arc lamps. There are some disadvantages related to mercury lamps such as being hazardous, easy to break, after-use disposal, short working shelf life and the possibility of gas leakage because of high thermal stress on the glass. The risks are higher when medium- and high-pressure UV lamps are used, which are operated at a high temperature range of 600 to 900 °C [119]. Much research is demonstrated to overcome these disadvantages. For this purpose, sunlight irradiation has been introduced as a replacement for mercury lamps at the laboratory scale for the potential applications of photo-Fenton processes [120,121]. Solar generative photo-Fenton reactions have demonstrated high performance, achieving a high degree of mineralization as well as a level of performance up to 90% within a short reaction time [122]. The pH of the solution is another important parameter affecting the performance of the photo-Fenton process, as it strongly influences the complex formation or leaching of the catalyst [123]. 

The Photo-Fenton process was reported as a comparatively efficient method for the degradation of various synthetic dyes [124,125,126]. For example, among many advanced oxidation processes applied for the degradation of RB-19 dye, the photo-Fenton process was the most efficient method, showing 94.5% dissolved organic carbon and 99.4% total color removal [127,128]. 

### 3.2. Electro-Fenton Process

In recent years, electrochemical technology has gained much attention for the removal of wastewater pollutants. Many research articles are available that give a detailed overview of the electro-Fenton process for the degradation of synthetic dyes in wastewater [129,130]. There are notable advantages of the electrochemistry, including energy efficiency, versatility and environmental suitability as the electron and main-stream reagents are clean. Hence, by coupling the electrochemistry with the Fenton process, the oxidation efficiency can be significantly improved [131]. The electro-Fenton oxidation process consists of either adding Fe^2+^ or reducing Fe^3+^ electrochemically along with the simultaneous production of H_2_O_2_ from the reduction of O_2_ on the electrodes [131]. Hydrogen peroxide is electrogenerated in acidic solutions by the two-electron reduction of oxygen on the cathode surface according to Equation (10) [132].

O_2_ + 2H^+^ + 2e^-^ → H_2_O_2_ (10)

In comparison with the conventional Fenton process, the major benefit of this indirect electro-oxidation approach is the higher degradation rate of the organic pollutants due to the continuous transformation of the Fe^3+^ to Fe^2+^ at the cathode according to Equation (11) [133].

Fe^3+^ + e^-^ → Fe^2+^ (11)

Fe^2+^ reacts with H_2_O_2_ to form active hydroxyl radicals in the aqueous medium. A continuous transformation of Fe^3+^ ensures the presence of sufficient Fe^2+^ ions which efficiently produce hydroxyl radicals, resulting in the higher degradation rate of the synthetic dyes [134]. Recently, a new technique was introduced for the electro-Fenton process in which a reduced graphene oxide (RGO) was electrochemically deposited on the surface of carbon felt and high performance was observed in the elimination of dyes, better stability and increased H_2_O_2_ formation. Table 2 summarizes the studies on the degradation of dyes by the electro-Fenton process. 

### 3.3. Sono-Fenton Process

In recent years, ultrasonic waves have been employed for the degradation of highly contaminated wastewater. Ultrasonic is a sound wave with a frequency of approximately 20 kHz or above, which is greater than the upper limit of the human hearing range. The use of ultrasonic energy creates alternating expansion and compression cycles. The expansion cycles of ultrasonic waves result in acoustic cavitation in the form of microbubbles [149]. Later on, these microbubbles build up to a certain size and collapse fiercely during the compression wave cycle, resulting in several hundreds of atmospheric pressure and a several thousand Kelvins of temperature that could be up to the range of 1000 atm and 5000 K, respectively [150,151]. This energy dispensation phenomenon of bubble creation and collapse is called cavitation or the cold boiling process. Although these intense conditions live for a short interval, the degradation of organic pollutants is achieved either by pyrolytic cleavage or the generation of hydroxyl radicals. Under these vigorous conditions, highly reactive species such as hydroxyl (**^∙^**OH) and hydrogen (H**^∙^**) radicals are formed as described in Equations (12)–(15) [25,26,27,76,77]. This sono-chemical oxidation process creates an oxidative environment by the implementation of ultrasonic waves in the aqueous phase.

H_2_O +))) → **^∙^**OH + H (12)

O_2_ +))) → 2 **^∙^**O (13)

O + H_2_O → 2 **^∙^**OH (14)

H**^∙^** + O_2_ → OH + **^∙^**O (15)

The combination of the ultrasonic and Fenton processes exhibits synergistic effects towards the degradation of organic pollutants because of the common fundamental oxidation mechanism [152,153]. The mechanism involves the reaction of H_2_O_2_ with Fe^2+^ ions to generate active hydroxyl radicals similar to the Fenton process, whereas the resulting Fe^3+^ ions react with H_2_O_2_ to generate an intermediate iron complex which dissociates into Fe^2+^ and **^∙^**OOH under the influence of ultrasound conditions as shown in Equation (16).

[Fe^III^(OOH)]^2−^ +))) → Fe^2+^ + **^∙^**OOH (16)

These Fe^2+^ ions further react with H_2_O_2_, resulting in the production of hydroxyl radicals. Therefore, the sono-Fenton process generates a higher concentration of hydroxyl radicals than that produced in the absence of ultrasonic waves. Hence, the combination of the ultrasonic and Fenton system (Fe^2+^/H_2_O_2_) is favorable and widely studied in detail in the literature [154]. From the literature, the sono-Fenton process is summarized as the high-performance process in terms of reaction rate and H_2_O_2_ usage. The self-production of oxidant species is favorable to overcome the extra cost of H_2_O_2_. However, the high energy consumption of ultrasonic systems restricted the implementation of sono-Fenton system-based technologies. The degradation of various dyes by sono-Fenton and sono-photo-Fenton systems are described in Table 3.

## 4. Non-Iron Metal Catalysts for Hydroxyl Radical-Based Oxidation

As the Fenton reaction using an iron-based catalyst has a significant drawback of a very narrow acidic pH region to attain the efficient decomposition of dyes [166], researchers focused on developing non-iron metal catalysts to overcome these shortcomings. It is suggested in various research works that transition metals other than iron, existing in at least two oxidation states such as Cu, Ru, Mn, Ag, and Co, can catalyze the formation of hydroxyl radicals from H_2_O_2_ [166,167,168,169,170]. There have been reports on the use of the colloidal nanoparticles of Au, Ag, and Pd for the degradation of methylene blue dye [171,172]. Among heterogeneous non-iron catalysts, Cu/Li_2_O/γ-Al_2_O_3_ [173], TiO_2_ nanoparticles on foamed polyethylene sheets [174], NiO/Al_2_O_3_ [175], etc. have also been applied to attain the efficient degradation of synthetic dyes in wastewater. Heterogeneous catalysts are considered more efficient and environmentally benign for catalytic application. 

Salem et al., applied the Cu–ethylenediamine complex, supported on clay montmorillonite K10, as a heterogeneous catalyst to degrade acid blue 29 (AB29) dye, using H_2_O_2_ as an oxidant [176]. Almost 88.2% of the decolorization was obtained at 40 °C within a reaction time of 18 min. The authors also reported the influential role of the concentrations of the reactants and the temperature on the efficiency for dye decomposition. The efficient Cu catalyzed decolorization of the dye solution was attributed to the formation of peroxo intermediates and hydroxyl radicals which acted as active oxidants to degrade AB dye.

Xaba et al. synthesized Pt nanoparticles in different sizes supported on mesoporous Co_3_O_4_ and applied them to attain the catalytic oxidative degradation of methylene blue (MB) dye, with H_2_O_2_ as an oxidant [177]. The highly efficient degradation of MB was achieved at ambient temperature conditions. Like other researchers, the authors reported different factors, including temperature, the concentration of dye, and H_2_O_2_, affecting catalytic activity for the dye decomposition process. An increase in temperature and H_2_O_2_ concentration resulted in a significant incline in catalytic activity, while declining activity occurred on increasing the initial concentration of dye in the reactant stream. Amini et al. fabricated a MgAl-LDH-supported polyoxomolybdate catalyst for the degradation of methylene blue (MB) and rhodamine B (RB) dyes separately [178]. The catalyst showed higher activity in the presence of H_2_O_2_ as an oxidant, giving almost 100% degradation of MB and RB within a reaction time of 60 and 80 min, respectively, at ambient conditions. The efficiency for the catalytic degradation of both of the dyes increased dramatically on the increasing concentration of H_2_O_2_. In contrast to iron-based catalysts, the alkaline medium was found to be more suitable to attain higher activity of the catalyst.

Among various supports, zeolites have also been recognized as an effective support for catalyst synthesis. Ag and Co ion-exchanged Y-type zeolites were synthesized and applied by Alekhina et al. for the catalytic degradation of carmoisine as an example of azo dyes [166]. They observed the highest oxidative degradation of dye using H_2_O_2_, specifically for CoNaY catalyst in a slightly alkaline medium. On continuing their research on metal ion-exchanged zeolites, in another paper, Alekhina et al. compared the catalytic activities of Fe or Co ion-exchanged HY and NaY zeolites for the decomposition of carmoisine dye at 60 °C [170]. The authors also studied the effect of catalyst preparation conditions on the efficiency of the reaction. According to their findings, the complete decolorization of the carmoisine solution was attained in alkaline and weekly acidic media using CoNaY as a catalyst. Whereas, FeHY as a catalyst was mostly effective in a weakly acidic medium. Hence, it can be suggested, by considering all the above mentioned studies on different catalysts, that suitable reaction conditions to attain efficient activity for dye decomposition depend on the type of metal and support material as well as on the methods used for catalyst preparation. 

## 5. Metal-Coated Tubular Reactors

We developed tubular reactors with inner walls coated with a thin metal catalyst layer and applied them to attain the efficient decomposition of synthetic dyes using high-pressure high-temperature water (HPHT-H_2_O) as a reaction medium [179,180]. Microtubular reactors offer advantages including a simple flow reaction system, excellent mass, and heat transfer properties, a large surface-to-volume ratio, and an enhanced reaction rate [181,182,183,184]. Non-catalytic flow reaction processing using HPHT-H_2_O has also been applied for the degradation of dyes [182,183,184,185] The properties of water vary on increasing temperature and pressure from a polar liquid to an approximately nonpolar fluid above critical temperature (374.8 °C) and pressure (22.13 MPa) conditions. HPHT-H_2_O provides advantages including a high thermal reaction rate, better dissolution of organic matters, low viscosity, excellent transport properties, etc. [185,186,187,188]. These properties of HPHT-H_2_O make it a good alternative for various reaction mediums. The application of a tubular reactor with inner walls coated with a thin layer of the metal catalyst using HPHT-H_2_O as a reaction medium not only provides the advantages of catalytic wet oxidation but also of HPHT-H_2_O which ensures the complete decomposition of synthetic dyes in a short residence time. Here, we will summarize the experimental setup and the results of this novel approach for the complete decomposition of synthetic dyes using a catalytic tubular reactor and HPHT-H_2_O as reported in our published papers [179,180].

**Figure 2:**
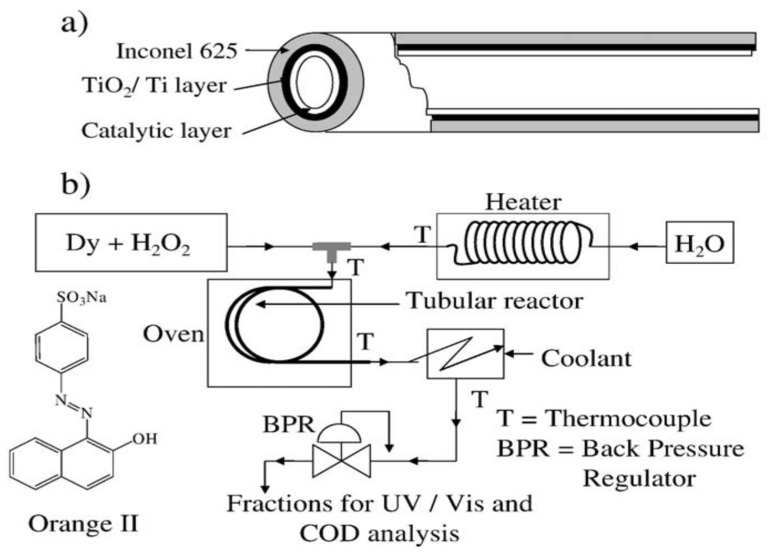
(**a**) Configuration of the catalyst-coated tubular reactor; (**b**) diagram of the HPHT-H_2_O flow reactor system [179].

The method for fabrication of catalytic tubular reactor is described in our various publications in detail [189,190,191,192,193]. Here, we provide a brief description. An Inconel (nickel alloy) tube (o.d. 1.6 mm, i.d. 0.5 mm, length 1000 mm) was used as reactor with an inlaid TiO_2_/Ti layer acting as a support for metal deposition. A thin layer of Pd as catalyst metal was coated on the inner walls of the reactor by an electroless plating technique. An electroless plating solution containing Pd precursor salt and reductant was continuously passed at constant temperature and flow rate to attain a thin deposited layer of Pd on the inner walls of the tubular reactor. The plating solution, after passing through the reactor, was analyzed by inductively coupled plasma-atomic emission spectroscopy (ICP-AES) to measure the amount of Pd deposited. Oxidation of the Pd surface to PdO was carried out by flowing air through the reactor at 750 °C for 2 h. This PdO-coated catalytic tubular reactor provided a remarkably high surface area-to-volume ratio of 0.8 × 10^4^ m^2^ m^−3^ [179,180]. Figure 2 presents the schematic diagram of the catalyst-coated tubular reactor (2a) and the experimental setup of the flow system (2b) [179]. Orange II dye in aqueous hydrogen peroxide (H_2_O_2_) solution was mixed with pre-heated water in the mixer and then passed continuously through the reactor at set temperature and pressure conditions. The solution from the reactor was analyzed for the removal of chemical oxygen demand (COD) and metal leaching. Continuous passage of the reactants mixed with HPHT-H_2_O from a reactor without metal coating resulted in 22.5% COD removal at 200 °C and 10 MPa gauge pressure, whereas, the application of a PdO-coated tubular reactor resulted in dramatically enhanced COD removal of 84.0% at the same reaction conditions. As reported by other researchers, the complete decomposition of synthetic dyes by oxygen or H_2_O_2_ requires much higher temperatures (above 400 °C) and longer reaction times (in minutes) [194]. Whereas, in our Pd catalyzed system, hydroxyl radicals generated by the catalytic decomposition of H_2_O_2_ [195,196,197] acted as a strong oxidant to destruct the stable aromatic ring of Orange II dye. Temperature played a vital role to attain higher efficiency for the catalytic decomposition of dyes, while the change in pressure did not notably affect catalytic activity. The almost complete removal of COD was obtained in a very short residence time of 4 s. Moreover, no leaching of metal was observed [179]. The initial concentration of the dye in the reactant stream also affected the rate of dye decomposition.

**Figure 3:**
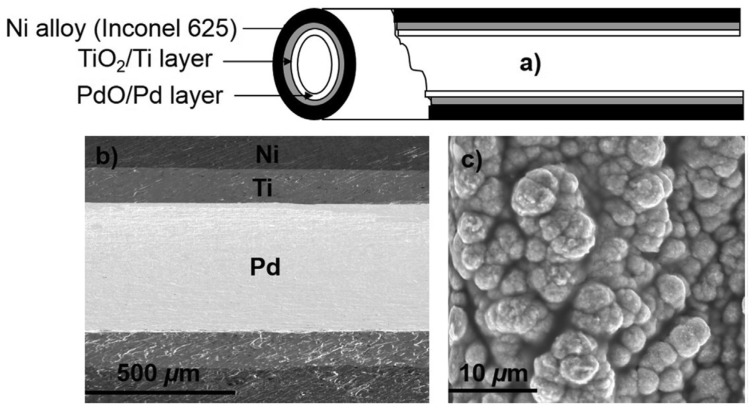
Images of the catalytic tubular reactor: (**a**) Schematic presentation of the tubular reactor; (**b**) Energy-dispersive X-ray spectroscopy (EDX) mapping of the longitudinal section of the Ni alloy (Inconel 625) tube with the TiO_2_/Ti secondary layer coated with the thin Pd layer; (**c**) Scanning electron microscopy (SEM) image of deposited Pd [180].

In another paper, we applied a catalytic tubular reactor using HPHT-H_2_O to decompose Remazol Brilliant Blue R (RBBR) as another example of synthetic dyes. RBBR is one of the most important synthetic dyes frequently used in the textile industry and as a starting material in the production of polymeric dyes. Figure 3 is adapted from our published paper [180]. Figure 3a shows the schematic diagram of a tubular reactor coated with a thin layer of the metal catalyst. The scanning electron microscopy (SEM) image (Figure 3b) presents the longitudinal section of the tubular reactor with thoroughly coated inner walls with the thin Pd layer. The magnified SEM image (Figure 3c) presents the round-shaped morphology of deposited Pd crystals. The experimental set up was similar, as mentioned earlier for the decomposition of orange II dye. The complete removal of total organic carbon (TOC) was attained at 300 °C and 10 MPa pressure within a short residence time of 3.2 s. We also studied the effects of temperature, pressure, the initial concentration of the dye and residence time on catalytic activity, which is explained in detail in a following section of this paper, “factors affecting catalytic activity”.

A catalytic flow reaction system using a tubular reactor coated with a thin layer of PdO provided a continuous and efficient approach to the complete removal of synthetic dyes. In contrast to the packed-bed reactor, our hollow tubular reactor coated with a thin layer of metal catalyst provided a smooth and continuous flow of reaction medium. Moreover, no deactivation or leaching of metal catalyst was observed. The durability and robustness enabled repeated use of the catalytic tubular reactor. This approach of tubular reactors coated with a thin layer of catalyst using HPHT-H_2_O provides an efficient technique for the complete removal of synthetic dyes within a very short residence time. Moreover, in contrast to other catalytic approaches, this process does not strictly depend on the pH of the reaction medium, which leads to the broad applicability of this technique. 

## 6. Sulfate Radical-Based Catalytic Oxidation

**Figure 4:**
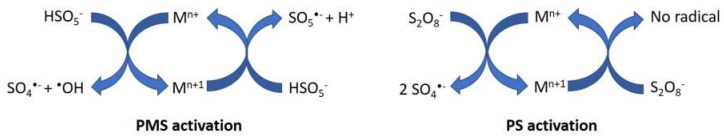
Transition metal-based catalytic activation of peroxymonosulfate (PMS) and persulfate (PS) [202].

Recently, the sulfate radical-based catalytic oxidation technique has attracted considerable attention for the decomposition of dyes in wastewaters. Sulfate radicals have been described as more efficient compared to hydroxyl radicals. For example, sulfate radicals possess a higher oxidation potential (2.5–3.1 V) [198,199,200,201,202] and react efficiently over a wide pH range (2–9) [198,199,200,201]. In addition, these radicals have a longer lifetime and react more selectively and efficiently by electron transfer with organic compounds containing unsaturated bonds or aromatic pi electrons [203,204,205]. In general, peroxymonosulfate (PMS) and persulfate (PS) are considered as oxidants to generate sulfate radicals [206]. The activation of PMS and PS is attained by various methods including heat, UV, ultrasound, or the use of a catalyst [201,206,207]. Several studies have reported the application of transition metal-based catalysts to activate PMS and PS. The catalytic activation of PS results in sulfate radicals, while that of PMS produces one hydroxyl and one sulfate radical [202]. In addition, PMS on reaction with the oxidized metal generates a sulfur pentoxide radical, which is less reactive than sulfate radicals but is capable of decomposing dye in wastewaters [202]. Figure 4, adapted from the published article [202], demonstrates the activation mechanism of PMS and PS by the catalyst.

Cobalt (Co) and silver (Ag) have proven to be the most effective transition metals for the activation of PMS and PS, respectively [201,202]. Mostly, the Co/PMS system has been studied for the removal of dyes. As homogeneous Co^2+^/PMS leads to secondary water pollution [198,199,208], heterogeneous cobalt/PMS systems were also introduced. These heterogeneous catalysts included carbon [209], C_3_N_4_ [210] or metal oxide supported catalysts [211,212]. Heterogeneous catalysts provide the advantage of less or no metal contamination and show superior catalytic activity to degrade the harmful dyes dispersed in water bodies. Shukla et al. synthesized cobalt ion-exchanged zeolites using ZSM-5, zeolite-A, and zeolite-X as supports where the highly efficient degradation of phenol was attained by CO-ZSM-5 [169]. Wang et al. prepared an Al_2_O_3_-based CoFe_2_O_4_ catalyst using a sol–gel method exhibiting high degradation efficiency of sulfachloropyridazine [213]. Hu et al. prepared nickel-foam supported Co_3_O_4_-Bi_2_O_3_ catalysts for bisphenol A (BPA) removal by peroxymonosulfate activation at room temperature conditions [198]. Over 91% of BPA was degraded in a pH range of 3.0–7.0 within a reaction time of 30 min. In contrast to the hydroxyl radical system, sulfate radical-based catalytic oxidation has the potential to attain high activity within a broad pH range which increases the applicability of this approach to the degradation of a wide variety of pollutants in industrial effluents. In another paper by these authors [199], they applied ZnCo_2_O_4_ catalyst/PMS to BPA removal. They prepared catalysts changing different variables, including microwave temperature, microwave duration, calcination temperature, and calcination duration. Under the conditions of [ZnCo_2_O_4_] as 0.2 gL^−1^ and [PMS]/[BPA]_molar_ as 2.0, a BPA degradation efficiency of 99.28% was obtained within 5 min. Over 98.21% of BPA was degraded within a pH range of 4.0–9.0. In all the above mentioned published papers, researchers have emphasized the suitability of the sulfate radical-based catalytic technique for the treatment of wastewater at a wide range of pH.

## 7. Factors Affecting Catalytic Activity

### 7.1. pH 

Generally, the quality of water is complex, and it exists at any pH value. Therefore, it is very important to determine the effect of the pH on the degradation efficiency of the catalytic system. Catalytic oxidation using H_2_O_2_ as an oxidant is influenced by the pH of the reaction medium [214]. The Fenton process is strongly dependent on the pH of the solution as it controls the production of hydroxyl radicals and the concentration of ferrous ions. An acidic medium is preferred for the decomposition of dyes using Fenton reagent [215,216]. The activity of Fenton reagent is reduced at higher pH due to the formation of relatively inactive iron oxohydroxides and ferric hydroxide precipitates [214,215,216], while a reaction medium with a highly acidic pH is also considered inefficient [217]. The plausible cause of reduced activity at very low pH is associated with the existence of iron complex species [Fe(H_2_O)_6_]^2+^, which reacts more slowly with H_2_O_2_ than other species [218]. Another assumption is solvation of the peroxide in the presence of a high concentration of H^+^ ions to form a stable oxonium ion [H_3_O_2_]^+^. Oxonium ions make H_2_O_2_ more stable and reduce its reactivity with ferrous ions [217]. Therefore, the efficiency of the Fenton process is reduced both at high and very low pH. For Fenton-like oxidation using various metals in addition to Fe, the efficiency for dye degradation also reduces with the increasing alkalinity of the solution [99,218,219,220]. This decline in efficiency is attributed to the rapid conversion of hydroxyl radicals to its less active conjugate base, ^•^O^−^ [221]. 

During our research on the catalytic decomposition of H_2_O_2_ using Pd or Pt-coated tubular reactors, we found that the catalytic conversion of H_2_O_2_ to hydroxyl radicals increased with increasing pH using oxidized Pd-coated tubular reactors at room temperature [191,222]. The optimized range of pH was described as 6–9. An increase in the acidity of the solution drastically decreased the decomposition of H_2_O_2_. On the other hand, the Pt-coated tubular reactor did not show any significant decrease in the conversion of H_2_O_2_ to hydroxyl radicals on reducing the pH of the solution. The oxidized Pd surface was supposed to be more susceptible to proton and/or anion interaction, thereby the access of H_2_O_2_ molecules must be suppressed, leading to the inhibition in decomposition, whereas the easy access of H_2_O_2_ molecules to the Pt surface increased its catalytic efficiency [191,193]. Hence, the suitable pH range varies from metal to metal used as a catalyst for the decomposition of H_2_O_2_.

A catalytic process using a sulfate radical as oxidant presents a system suitable for the dye decomposition at a broader range of pH than that using a hydroxyl radical [198,199,200,201,202]. However, a highly alkaline medium decreases the degradation efficiency. This phenomenon is suggested due to the excessive formation of OH¯, which generates hydroxyl radicals by consuming sulfate radicals. The weak oxidative ability and non-selectivity of hydroxyl radicals along with a decrease in the concentration of sulfate radicals decrease the efficiency for dye decomposition [202]. Therefore, it is required to optimize the pH of the reaction medium, which depends not only on the catalyst but is also highly influenced by the type of oxidant used in the process. 

### 7.2. Temperature

It is reported in many research papers that an increase in operational temperature could be beneficial for both the oxidation rate and the extent of the catalytic decomposition of synthetic dyes [218,222], whereas very limited research has been conducted to evaluate the influence of experimental temperature conditions on the performance of the catalyst for the decomposition of synthetic dyes. Mostly, Fenton-based methods have been carried out at room temperature [223]. Zazo et al. [223] reported a considerable improvement in the decomposition of phenol (a primary component of most of the synthetic dyes) by Fenton oxidation at a relatively higher temperature, where a decomposition efficiency of almost 80% was achieved at 120 °C, which declined to 28% on decreasing the experimental temperature to 25 °C. The authors also demonstrated an enhanced iron-catalyzed H_2_O_2_ decomposition into radicals at a higher temperature. Salem et al., also investigated the effect of temperature on the decolorization efficiency of acid blue 29 dye using a heterogenous Cu catalyst, keeping the concentrations of the dye, H_2_O_2_ and the catalyst constant [176]. They noticed an increase in the decolorization efficiency from 51.8 to 88.2% on increasing the operating temperature from 20 to 40 °C within 18 min of reaction time. 

The effect of temperature can also be associated with enhancing efficiency within a short residence time while keeping all other parameters fixed. During our research on the catalytic decomposition of synthetic dyes using a PdO-coated tubular reactor and HPHT-H_2_O as reaction medium along with H_2_O_2_ as an oxidant, we observed that the reaction was strongly dependent on temperature [179,180]. While using a reaction solution of Orange II (a synthetic dye), COD removal of 84.0% was attained at 200 °C, which increased to 99.0% on increasing experimental temperature to 300 °C within a residence time of 4 s and at a fix gauge pressure of 10 MPa [179]. Likewise, in another study on a solution of 20 ppm Remazol Brilliant Blue R dye, TOC removal enhanced sharply from 89 to 92 and 99.9% on increasing temperature from 200 to 250 and 300 °C, respectively, at 10 MPa pressure and a short residence time of 3.2 s [180]. Therefore, the catalytic decomposition of synthetic dyes at comparably higher temperatures provides a way to enhance the activity by a significant improvement of the oxidation rate and mineralization percentage within a fixed reaction time.

The catalytic process using a sulfate radical as an oxidant is also mostly studied at ambient temperature conditions [198,199,200,201,202] without considering the effect of operating temperature. Hence, the importance of temperature as a parameter in the catalytic decomposition of dyes cannot be denied. Therefore, a detailed investigation of the influence of temperature is highly required.

### 7.3. The Concentration of the Oxidant

The concentration of the oxidant plays a crucial role in the overall efficiency of the catalytic degradation process of dyes [126]. It is observed that the efficiency for the degradation of the synthetic dyes increased with an increase in the concentration of H_2_O_2_ in the reaction stream [224,225,226,227]. The steady-state concentration of hydroxyl radicals depends on the concentration of H_2_O_2_ and Fe^2+^ in the Fenton oxidation process. Tian et al., [228] reported that the color and COD removal increased to 94 and 50.7%, respectively, as the H_2_O_2_ increased to 125 mg/L in the Fenton process, while a further increase in the concentration of H_2_O_2_ decreased removal efficiency [228]. Similar results were also reported by other researchers [229]. The decrease in efficiency for dye decomposition on increasing H_2_O_2_ above the optimized concentration was attributed to the development of competition for adsorption on the surface of the catalyst, where the excessive H_2_O_2_ limits the access of the dye molecules. Besides, excessive H_2_O_2_ could reduce hydroxyl radicals as a radical scavenger [229,230]. Therefore, optimization of the H_2_O_2_ concentration in the catalytic oxidation process is highly important. As a study on iron-free catalytic oxidation, Salem, et al. examined the effect of H_2_O_2_ concentration on reaction rate for the decomposition of acid blue 29 dye using a Cu-based catalyst by maintaining the temperature, amount of catalyst and dye constant [223]. They reported an increase in decolorization efficiency from 26.6 to 84.3% on increasing H_2_O_2_ concentration from 0.02 to 0.4 M within 15 min. They attributed their findings to the enhanced generation of peroxo-intermediate or hydroxyl radicals on the increasing concentration of H_2_O_2_ in the reaction medium. The generation of peroxo-radicals in Cu-facilitated oxidation reactions were also reported by other researchers [231,232]. However, optimization for the required concentration of H_2_O_2_ should be conducted depending on the concentration of the synthetic dyes to be decomposed as the remaining concentration of unused H_2_O_2_ contributes to COD and is harmful to many of the organisms [226,227].

### 7.4. The Initial Concentration of Dye 

The initial concentration of dye plays an important role in practical applications. In general, a lower initial concentration is favored [23,226,231] to attain the efficient and complete decomposition of the dyes. As the effluent released from industries contains a very high concentration of the dye contents, dilution is required before proceeding for catalytic treatment regardless of the type of oxidant used [23,176,226]. Salem et al., observed a significant decrease in the efficiency for dye decolorization from 92.8 to 78.2% on the increasing concentration of acid blue 29 dye from 1×10^−4^ to 2×10^−5^ M, while working with a heterogenous Cu catalyst and keeping the concentration of H_2_O_2_ constant (0.2 M) [223]. During the application of an oxidized Pd-coated tubular reactor to the decomposition of synthetic dyes using a fixed concentration of H_2_O_2_ as an oxidant at high temperature and pressure conditions, we observed that an increase in the initial concentration of synthetic dye decreased the efficiency for decomposition [179,180]. At higher dye concentrations, the generation of hydroxyl radicals on the surface of the catalyst was suggested to be reduced since the active sites of the catalyst might be occupied by the dye molecules. An increased number of dye molecules and insufficient concentration of the active radicals decreased the efficiency of the decomposition process [176,179,180,224]. In contrast, Hassan et al. reported an increase in dye removal efficiency with increasing initial dye concentration [224,225]. This fact was associated with an increase in the probability of collision between the dye molecules and the oxidizing species on increasing the dye concentration in the reaction medium. However, the selection of the initial concentration of the dyes in reactants is also dependent on the concentration of the oxidizing agent, catalyst, and reaction/residence time of the process. For example, the initial concentration of synthetic dye is assessed by the amount of Fenton’s reagent used in the process [227]. 

### 7.5. Reaction Time

Reaction/residence time is an important parameter influencing the catalytic dye decomposition process regardless of the type of catalyst (homogeneous or heterogeneous). Generally, the rate of the decomposition reaction increases with an increase in the duration or residence time of a reaction, while keeping all other factors, e.g., pH, temperature, concentrations of dye, oxidant and the catalyst constant. Soraya Mohajeri et al. reported the effect of reaction time on the Fenton process [233]. They varied the reaction time from 30 to 120 min and observed increased COD and color removal from 45 to 69%. S. Karthikeyan et al. also noticed a linear increase in the removal of COD within 4 h of the homogeneous Fenton oxidation reaction, which slowed down on increasing the reaction time for a further 2 h [234]. They attributed this initial linear increase in COD reduction to the chemical oxidation of the dissolved organics in wastewater with hydroxyl radicals. The authors also confirmed the higher efficiency of heterogeneous Fenton oxidation compared to the homogenous process. An overall COD removal of 90% of the textile wastewater was obtained for a heterogenous catalyst in approximately 4 h, whereas a COD removal of 50% was attained for the homogeneous catalyst within a reaction time of 6 h.

Usually, a long reaction time in hours is required to attain a significantly increased COD and color removal, whereas the decomposition of dyes with an efficiency above 99% of COD removal can be obtained within a few seconds using a thin metal catalyst-coated tubular reactor and HPHT-H_2_O as the reaction medium [179,180]. The reaction time depends on other factors, including the temperature and concentration of the reacting species, etc. During our research on the catalytic decomposition of synthetic dyes using a PdO-coated tubular reactor, we observed that 89% TOC removal of Remazol Brilliant Blue R was obtained at 200 °C within a residence time of 3.9 s, which increased to 92 and 99.9 at 250 °C and 300 °C within a residence time of 3.6 and 3.2 s, respectively [180].

## 8. Conclusions

Dyes are one of the major pollutants of our environment. If these dyes are not removed from industrial effluents before entering into the aquatic system, this could be very harmful to all species on earth. The conventional methods are not very efficient in treating industrial wastewaters containing higher concentrations of synthetic dyes due to their recalcitrant nature and resistance to biodegradation. Catalytic oxidation is one of the advanced oxidation processes and is considered environmentally friendly and highly efficient for the degradation of dyes. The total mineralization of dyes is achieved in many processes. The Fenton reaction treatment is known to be very useful in the removal of dyes from wastewater using iron-based catalysts. Research efforts have also been focused on the establishment of iron-free catalytic systems using various other metals for the activation of H_2_O_2_. Tubular reactors with inner walls coated with a thin layer of metal catalysts have also been applied and found to be an efficient method for dye decomposition within a short residence time, i.e., seconds. Sulfate radical-based oxidation provides another efficient approach to the catalytic degradation of synthetic dyes.

The most challenging issue in the catalytic oxidation of synthetic dyes is the optimization of various reaction parameters as most catalytic processes depend heavily on various factors including pH, temperature, the concentration of the oxidant, the initial concentration of dyes and the reaction time. There exists an optimal value for almost every parameter depending on the type of catalyst and the oxidant to be used. By optimizing different factors appropriately, catalytic activity can be enhanced to the greatest extent, making this one of the most advanced oxidation processes for dye degradation.

## Figures and Tables

**Table 1:** Selected synthetic dyes commonly used in the textile industry: their types, applications, and hazardous effects.

Dye Pollutant	Application	Hazardous Effect	References
Aniline Yellow or 4-phenylazoaniline	Chemical industry, printer’s ink, intermediate for dye synthesis	Induces liver and epidermal tumors, high hepato-carinogenicity to male mouse	[43,44]
Benzamine (BZ)-based azo dye	Chemical industry	Carcinogenic effect on human urinary bladder and reported tumorigenic effect on laboratory animals	[45]
o-Aminoazotoluene (C.I. Solvent Yellow 3)	Food and chemical industry	Tumors in urinary bladder, gall bladder, lung, and live	[46]
Methyl Yellow (Butter Yellow) and derivatives	Chemical, food and textile industry	Highly toxic cancer-causing agent	[47]
Reactive Brilliant Red	Textile, paint industry	Inhibit function of human serum albumin, may react to body protein or enzyme	[48]
Sudan azo dye (1-phenylazo-2-naphthol)	Petrochemical, textile and food industry	Carcinogenic in nature	[49]
Benzidine and its congener	Chemical industry	Carcinogenic to human urinary bladder, pancreas, liver, gallbladder, bile duct, lung, large intestine, stomach and renal cell	[50]
Direct Blue 15 (dimethoxybenzidine-based dye)	Biological and staining applications	Poisonous effect and mutagenicity in reduction process, carcinogenic	[42,51]
p-phenylenediamine (p-PDA)	Hair dye, personal care	Possibility of bladder cancer and skin allergy	[52]
p-Nitroaniline	Dyes intermediate, antioxidants, pharmaceuticals, corrosion inhibitor, petrochemical	Mutagenic, human carcinogen and induces tumors	[53]
Acid Violet 7	Food, paint, paper, cosmetic, and especially in textile industries	Chromosomal aberration, acetylcholinesterase activity inhibition, membrane lipid peroxidation	[54]
o-Toluidine (2-methylaniline)	Intermediate for dye, rubber, and pharmaceuticals	Urinary bladder cancer	[55]
2, 4-Diaminotoluene	Dye industry	Induces tumor in rats and mice, potential human carcinogenic effect	[56]
Malachite Green	Dye stuff in silk, leather, paper and antimicrobial in aquaculture	Carcinogenic, mutagenic, chromosomal fractures, respiratory toxicity	[57]
2-Nitro-p-phenylenediamine	Chemical and pharmaceutical	Reported carcinogenic for female mice	[58]
2-Amino-4-nitrophenol	Cosmetic industry	Causes renal tubular cell hyperplasia	[59]
4-Nitro-o-phenylenediamine	Hair dye, cosmetic industry	Carcinogen to humans	[60]
Reactive Black 5 (sulfonated azo dye)	Color and dye industry	Restrict nitrogen use efficiency of plant, decrease the urease activity, carcinogenicity	[61,62,63]
o-Phenylenediamine (o-PDA)	Pharmaceutical, cosmetic products and corrosion inhibitor	Genotoxic, asthma, gastritis, rise in blood pressure, vertigo, tremors, and comas	[64]
Disperse Red 1 and Disperse Red 13	Textile industry	Mutagenic to salmonella with possibility on human beings, affecting the activity and composition of microbial communities	[65,66,67]
m-Phenylenediamine (m-PDA)	Dye component, additive for resin, coatings, polymers, cosmetic industry	Oxidation products are highly mutagenic	[68]
Congo Red	Cotton dyeing, textile industry	Carcinogenic and mutagenic	[69]
Nitro-group with monocyclic aromatic amines	Various chemical industries	Likely to be carcinogenic	[70]

**Table 2:** Degradation of dyes by an electro-Fenton process in various studies.

Dye Pollutant	References
Direct Orange 16	[135]
Acid Red 14	[136]
Basic Blue 3	[137]
4-Amino-3-hydroxy-2-p-tolylazo-naphthalene-1-sulfonic acid	[138]
Alizarin red	[139]
Yellow 52	[140]
4-Nitrophenol	[141]
Methyl Orange	[142]
Orange G	[143]
Rhodamine B	[144]
Lissamine Green B	[145]
Azure B	[145]
Reactive Black 5	[146]
Reactive Red 120	[147]
Orange II	[148]

**Table 3:** Degradation of wastewater pollutants by sono-Fenton and sono-photo-Fenton processes.

Dye Pollutant	References
Methylene Blue and Congo Red dyes	[155]
Reactive Blue 69	[156]
Aromatic Amines	[157]
Reactive Blue	[158]
Cephalexin	[159]
Non-volatile organic compound, dyes, Carbofuran	[160]
Bisphenol A	[161]
5-Fluorouracil	[162]
Nitrobenzene	[163]
Rhodamine B dye	[164]
Azure B	[165]
